# Norepinephrine alleviates cyclosporin A-induced nephrotoxicity by enhancing the expression of SFRP1

**DOI:** 10.1515/med-2023-0769

**Published:** 2023-08-14

**Authors:** Huaibin Sun, Zhiguo Peng, Kao Liu, Shengli Liu

**Affiliations:** Department of Organ Transplantation, Qilu Hospital of Shandong University, No. 107, Wenhuaxi Road, Jinan, Shandong, 250012, China; Department of Organ Transplantation, Qilu Hospital of Shandong University, Jinan, Shandong, 250012, China

**Keywords:** nephrotoxicity, cyclosporin A, norepinephrine, secreted frizzled-related protein 1

## Abstract

Norepinephrine (NE) has a certain effect on the improvement of renal function. However, whether NE can alleviate cyclosporin A (CsA)-induced nephrotoxicity needs further study. The effect of CsA (1.25, 2.5, 5, and 10 μM) on the human renal epithelial cell vitality, lactate dehydrogenase (LDH) activity, apoptosis, and secreted frizzled-related protein 1 (SFRP1) level was examined by cell counting kit-8, enzyme-linked immunosorbent assay, flow cytometer, and western blot. The effect of NE on the LDH activity, apoptosis, and SFRP1 level of human renal epithelial cells induced by CsA was examined again. After silencing of SFRP1 in human renal epithelial cells, the SFRP1 level, cell vitality, and apoptosis were examined again. CsA (1.25, 2.5, 5, and 10 μM) attenuated the cell vitality and SFRP1 level but enhanced the LDH activity and apoptosis in human renal epithelial cells, while the above effects were reversed by NE. Moreover, SFRP1 silencing reversed the regulation of NE on the SFRP1 level, cell vitality, and apoptosis in human renal epithelial cells induced by CsA. In conclusion, NE relieved CsA-induced nephrotoxicity via enhancing the expression of SFRP1.

## Graphical abstract

**Figure:**
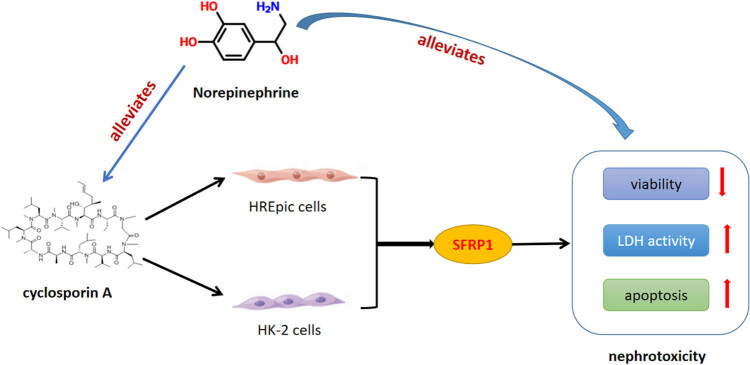

## 1 Introduction

In the past 30 years, calcineurin inhibitors represented by cyclosporin A (CsA) have been the first-line drugs for anti-rejection therapy after kidney transplantation, and have also been widely used in the treatment of a variety of autoimmune diseases [1]. CsA notably ameliorated the short-term survival rate of patients with organ and bone marrow transplantation and lessened the recurrence rate and mortality of immune diseases [2]. However, with the expansion of the scope of clinical application and the extension of medication time, its adverse reactions have been gradually revealed. The adverse reactions of CsA are usually dose-related; reducing dose can alleviate the symptoms of adverse reactions, but increase the probability of rejection, which limits the clinical application of CsA [3]. The adverse reactions caused by CsA include hepatotoxicity, nephrotoxicity, neurotoxicity, and severe hypertension, of which nephrotoxicity is considered to be the most important adverse reaction, causing serious damage to the renal function and structure of patients [4–6]. Therefore, it is an urgent problem to find a method that can maintain the concentration of CsA within the application range while reducing its nephrotoxicity.

Norepinephrine (NE) is an alpha-adrenergic receptor agonist, which can effectively contract peripheral blood vessels and is commonly used in the treatment of hypotension [7]. Traditionally, NE has been considered unsuitable for renal transplantation because of concerns about constriction of renal vessels and reduction of renal blood flow. However, a study has manifested that intravenous infusion of small doses of NE could increase cardiac output and renal perfusion pressure [8]. Deruddre et al. reported that in patients with severe infection, the levels of the renal cortical interlobular artery and intralobular artery presented a low perfusion state, while NE treatment increased the mean arterial pressure from 65 to 75, the resistance index of the renal interlobular artery in the renal cortex notably raised, and the renal function obviously improved [9]. These studies clarified that NE has a certain effect on the improvement of renal function. However, whether NE can alleviate CsA-induced nephrotoxicity needs further study.

We preliminarily analyzed the dataset GSE111516 and obtained the abnormal gene expression in the kidney of mice treated with daily CsA for 6 weeks [10]. We searched the literature for the first five genes and discovered that the expression of secreted frizzled-related protein 1 (SFRP1) was associated with multiple kidney diseases, including renal cell carcinoma [11], obstructive nephropathy [12], and diabetic nephropathy [13]. Moreover, Hawkshaw et al. discovered that CsA could notably restrain the expression of SFRP1 in the dermal papilla of human scalp hair follicles [14]. Additionally, Lin et al. discovered that NE-induced HSCs secreted SFRP1 to intensify HCC development via enhancing the autocrine feedback loop of the Wnt16b/β-catenin pathway [15]. However, whether NE can alleviate CsA-induced nephrotoxicity by modulating SFRP1 has not been reported yet.

Collectively, we aim to explore the function of CsA on two human renal epithelial cells (HREpic and HK-2), and whether NE can alleviate CsA-triggered nephrotoxicity by modulating SFRP1.

## 2 Materials and methods

### 2.1 Cell culture

HREpic cells (#4120, ScienCell, USA) were maintained in an epithelial cell medium (#4101, ScienCell, USA). HK-2 cells (C1116, WHELAB, China) were grown in Dulbecco’s modified eagle’s medium complete medium (M0101A, WHELAB, China). The above cells were placed in a 37℃, 5% CO_2_ cell incubator (BB150, Thermo Fisher, USA).

### 2.2 Cell transfection

Small interfering RNA targeting secreted frizzled-related protein 1 (siSFRP1, siG000006422C-1-5) and its negative control (siNC, siN0000001-1-5) were obtained from Ribobio (China). We used the ViaFect™ transfection reagent (E4981, Promega, USA) to transfect siNC and siSFRP1 into HREpic and HK-2 cells. The transfection efficiency was examined after 48 h of the transfection.

### 2.3 Experimental design

First, CsA (abs47032299, Absin, China) was dissolved in ethanol (E111991, aladdin, China) and then diluted in medium without fetal bovine serum to the dilution of 1.25, 2.5, 5, and 10 μM. We used the diluted CsA to stimulate cells for 24 h. Second, to explore the function of NE (A9512, Sigma-Aldrich, USA) on nephrotoxicity induced by CsA, cells were assigned to the control group (cells without any drug treatment were treated as control), NE group (cells were subjected to 10 μM NE for 24 h) [16], CsA group (cells were stimulated with 5 μM CsA for 24 h), and CsA + NE group (cells were stimulated with 5 μM CsA for 24 h and then reacted with 10 μM NE for 24 h). In the third part of the cell experiment, cells were divided to the control group, CsA group, CsA + NE group, CsA + NE + siNC/CsA + NE + siSFRP1 group (cells transfected with siNC or siSFRP1 were stimulated with 5 μM CsA for 24 h and then reacted with 10 μM NE for 24 h).

### 2.4 Cell viability assay

We used cell counting kit-8 (CCK-8; CCK02, GEFAN, China) to determine cell vitality. Based on the above experimental design, cells (2 × 10^4^) were treated and then cultivated in the cell incubator for 48 h. Thereafter, we used 10 μL CCK-8 solution to treat the cells. They were placed in the cell incubator for 3 h. In the end, the absorbance (450 nm) was estimated using a microplate reader (Infinite M200, Tecan, Austria).

### 2.5 Lactate dehydrogenase (LDH) detection

A human LDH kit (BPE10891, Lengton, China) was used in this research. Briefly, treated cells (2 × 10^4^) were centrifuged to gain the cell supernatant. Each sample hole was added to a 40 μL cell supernatant followed by the addition of 10 μL anti-LDH antibody and 50 μL streptavidin-HRP. After covering with the sealing film, we gently vibrated the reaction wells and incubated them at 37℃ for 60 min. After washing, each hole was first added to 50 μL developer A and then added to 50 μL developer B. After mixing, they were developed at 37℃ away from light for 10 min. They were then reacted with a 50 μL stop solution. Finally, we used the microplate reader to evaluate the absorbance at 450 nm.

### 2.6 Apoptosis assay

The apoptosis of treated cells was examined using the Annexin V-FITC/propidium iodide (PI) kit (CA1020, Solarbio, China). After treatment, cells (1.5 × 10^6^) were harvested and washed. We used 1× binding buffer to re-suspend the cells so that the cell density reached 1 × 10^6^ cells/mL. Next, each hole was added to 100 μL cells (1 × 10^5^ cells) followed by the addition of 5 μL Annexin V-FITC. They were then incubated at 37℃ away from the light for 10 min. After adding 5 μL PI, they were placed in a dark room at 37℃ for 5 min. Finally, we used a flow cytometer (C6, BD, USA) to examine the FITC and PI signals.

### 2.7 Western blot

The treated cells were routinely harvested and lysed by a cell lysis buffer (abs9225, Absin, China) [17]. After centrifugation, the protein concentration of the cell supernatant was examined using a BCA kit (abs9232, Absin, China). After denaturation, equal quantities of protein were electrophoresed and then transferred onto a polyvinylidene difluoride membrane (#ISEQ00010, Merck-Millipore, USA). After blocking, they were incubated with primary antibody overnight at 4℃ followed by the addition of secondary antibody (S0001, Affinity, USA) for 1 h at 37℃. Finally, protein expression was examined with a color reagent (abs920, Absin, China) in a chemic luminous instrument gel imaging system (A44114, Invitrogen, USA). Glyceraldehyde-3-phosphate dehydrogenase (GAPDH) was employed for the housekeeping gene. The primary antibodies of SFRP1 (1:1,000, 35 kDa, #3534) and GAPDH (1:1,000, 37 kDa, #2118) were obtained from CST (USA).

### 2.8 Quantitative real-time polymerase chain reaction (qRT-PCR)

First, we adopted Lezol (NR0003, LEAGENE, China) to acquire RNA from treated cells. Next, the qRT-PCR reaction was performed using a TaqMan One Step RT-qPCR kit (T2210, Solarbio, China) in a PCR system (QuantStudio 5, ABI, USA). GAPDH was employed for the internal controls and data were expressed as 2^−ΔΔCt^ method [17]. The primer sequences were listed 5′ to 3′: SFRP1, (F) ACGTGGGCTACAAGAAGATGG; (R) CAGCGACACGGGTAGATGG; GAPDH, (F) TGTGGGCATCAATGGATTTGG; (R) ACACCATGTATTCC GGGTCAAT.

### 2.9 Statistical analysis

Data were gathered and analyzed using Graphpad Prism 8.0 (GraphPad Software Inc., USA). All data were shown as mean ± standard deviation (SD). An independent sample *t-*test was employed between groups. Multiple groups were done through a one-way analysis of variance followed by Tukey’s *post hoc* test. *P* < 0.05 was considered significant.

## 3 Results

### 3.1 CsA attenuated the cell vitality but enhanced the LDH activity and apoptosis in HREpic and HK-2 cells

**Figure 1:**
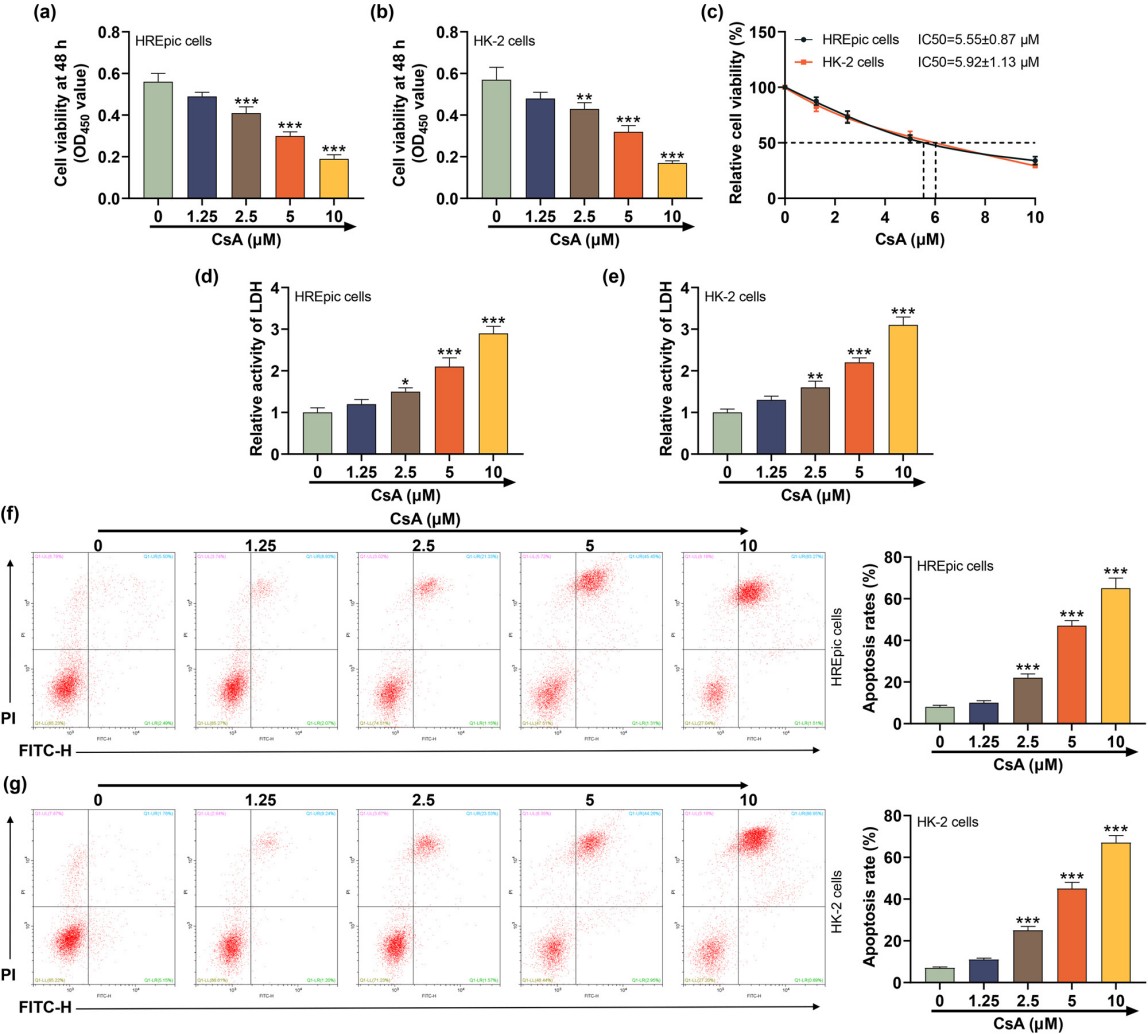
CsA attenuated the cell vitality and SFRP1 level but enhanced the LDH activity and apoptosis in HREpic and HK-2 cells. (a) and (b) Effect of CsA on the cell vitality in HREpic and HK-2 cells was examined by CCK-8. (c) Half maximal inhibitory concentration (IC_50_) of CsA in HREpic and HK-2 cells. (d) and (e) Effect of CsA on the LDH activity in HREpic and HK-2 cells was examined by enzyme-linked immunosorbent assay (ELISA). (f) and (g) Effect of CsA on apoptosis in HREpic and HK-2 cells was examined by flow cytometer. All experiments have been performed in triplicate and data were expressed as mean ± SD.^*^ *P* < 0.05, ^**^ *P* < 0.01, ^***^ *P* < 0.001 vs 0 μM. CsA concentrations: 1.25, 2.5, 5, and 10 μM.

We first used 1.25, 2.5, 5, and 10 μM CsA to stimulate cells and confirmed that CsA (2.5, 5, and 10 μM) manifested a notable inhibitory effect on cell vitality of HREpic and HK-2 cells in a dose-dependent manner (Figure 1a and b, *P* < 0.01). The half maximal inhibitory concentration (IC_50_) of CsA in HREpic cells was 5.55 ± 0.87 μM and the IC_50_ of CsA in HK-2 cells was 5.92 ± 1.13 μM (Figure 1c). Next, the result showed that CsA (2.5, 5, and 10 μM) evidently elevated the LDH activity of HREpic and HK-2 cells, and there was a dose-dependent effect (Figure 1d and e, *P* < 0.05). Meanwhile, the effect of CsA on the apoptosis of HREpic and HK-2 cells was detected, and we discovered that CsA (2.5, 5, and 10 μM) promoted the apoptosis of HREpic and HK-2 cells (Figure 1f and g, *P* < 0.001).

### 3.2 CsA inhibited SFRP1 level in HREpic and HK-2 cells

**Figure 2:**
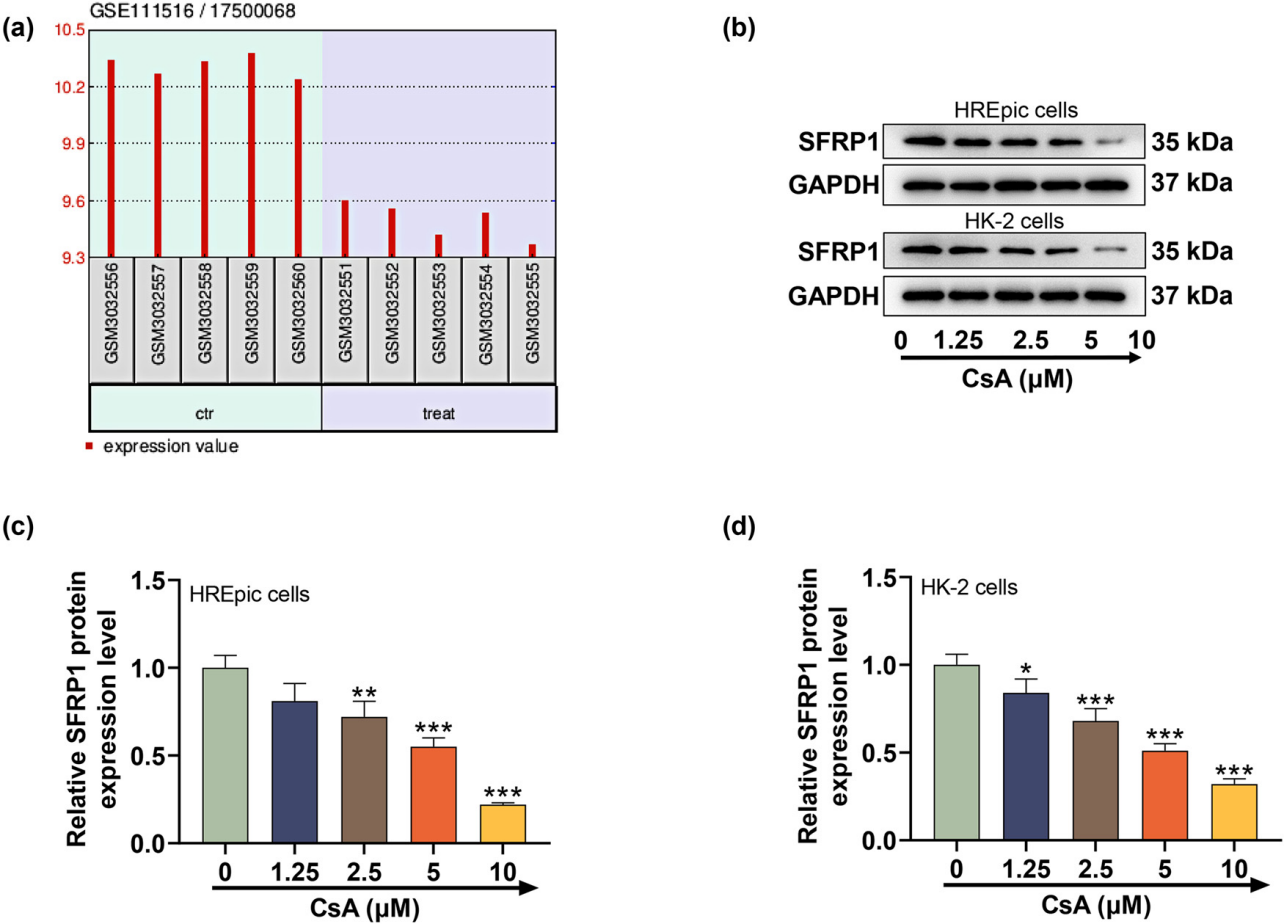
CsA inhibited SFRP1 level in HREpic and HK-2 cells (a) Expression of SFRP1 was analyzed based on the dataset GSE111516. (b)–(d) Effect of CsA on SFRP1 level in HREpic and HK-2 cells was examined by western blot. GAPDH was served as an internal control. All experiments have been performed in triplicate and data were expressed as mean ± SD.^*^ *P* < 0.05, ^**^ *P* < 0.01, ^***^ *P* < 0.001 vs 0 μM. CsA concentrations: 1.25, 2.5, 5, and 10 μM.

Based on the dataset GSE111516 analysis, SFRP1 expression was lower in the kidneys of CsA treated mice than that in control mice (Figure 2a). Moreover, the SFRP1 protein level of HREpic and HK-2 cells was notably restrained by CsA (2.5, 5, and 10 μM) and 1.25 μM CsA also evidently attenuated the SFRP1 level of HK-2 cells (Figure 2b–d, *P* < 0.05).

### 3.3 NE partially offset the regulation of CsA on the LDH activity, apoptosis, and SFRP1 level in HREpic and HK-2 cells

**Figure 3:**
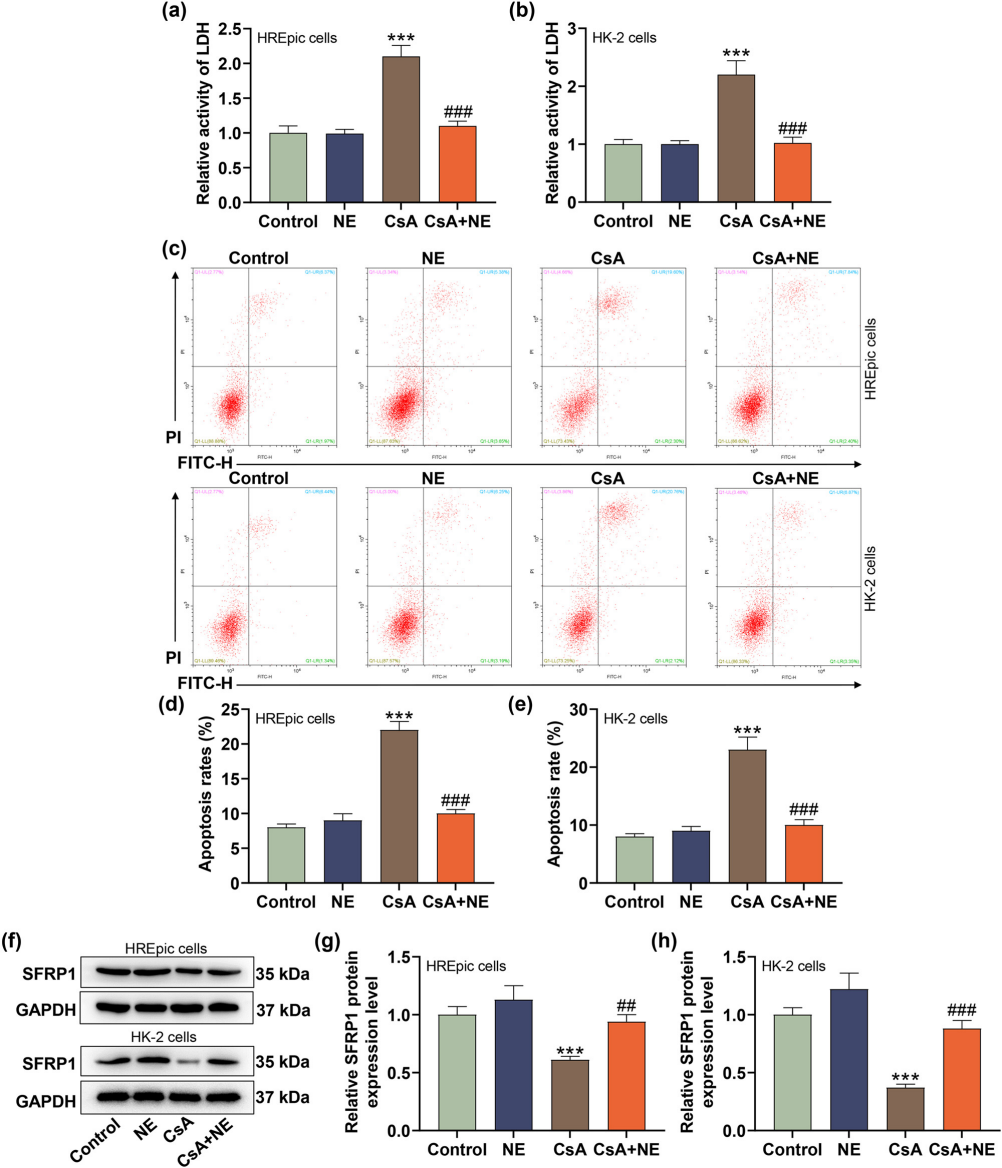
NE partially offset the regulation of CsA on the LDH activity, apoptosis, and SFRP1 level in HREpic and HK-2 cells. (a) and (b) Effect of NE on the LDH activity of human renal epithelial cells induced by CsA was examined by ELISA. (c)–(e) Effect of NE on apoptosis of human renal epithelial cells induced by CsA was examined by flow cytometer. (f)–(h) Effect of NE on the SFRP1 level of human renal epithelial cells induced by CsA was examined by western blot. GAPDH was served as an internal control. All experiments have been performed in triplicate and data were expressed as mean ± SD. ^***^ *P* < 0.001 vs control; ^##^ *P* < 0.01, ^###^ *P* < 0.001 vs CsA. CsA concentration: 5 μM; NE concentration: 10 μM.

In this part of the study, we further analyzed the effect of NE on renal cells induced by CsA. We chose 5 μM CsA for subsequent analysis, as displayed in Figure 3a and b, the LDH activity of HREpic and HK-2 cells was higher in CsA group than that in control group (*P* < 0.001), while NE treatment had no significant effect on the LDH activity; the promoting effects of CsA on the LDH activity of HREpic and HK-2 cells were reversed by NE (*P* < 0.001). In addition, CsA promoted the apoptosis of HREpic and HK-2 cells, while NE treatment reversed the promoting effects of CsA on the apoptosis of HREpic and HK-2 cells (Figure 3c–e, *P* < 0.001). The western blot assay illustrated that NE treatment had no significant effect on the SFRP1 protein level of HREpic and HK-2 cells, while it partially reversed the inhibiting effects of CsA on the SFRP1 protein level of HREpic and HK-2 cells (Figure 3f–h, *P* < 0.01).

### 3.4 Silencing of SFRP1 reversed the regulation of NE on the SFRP1 level, cell vitality, and apoptosis in HREpic and HK-2 cells induced by CsA

**Figure 4:**
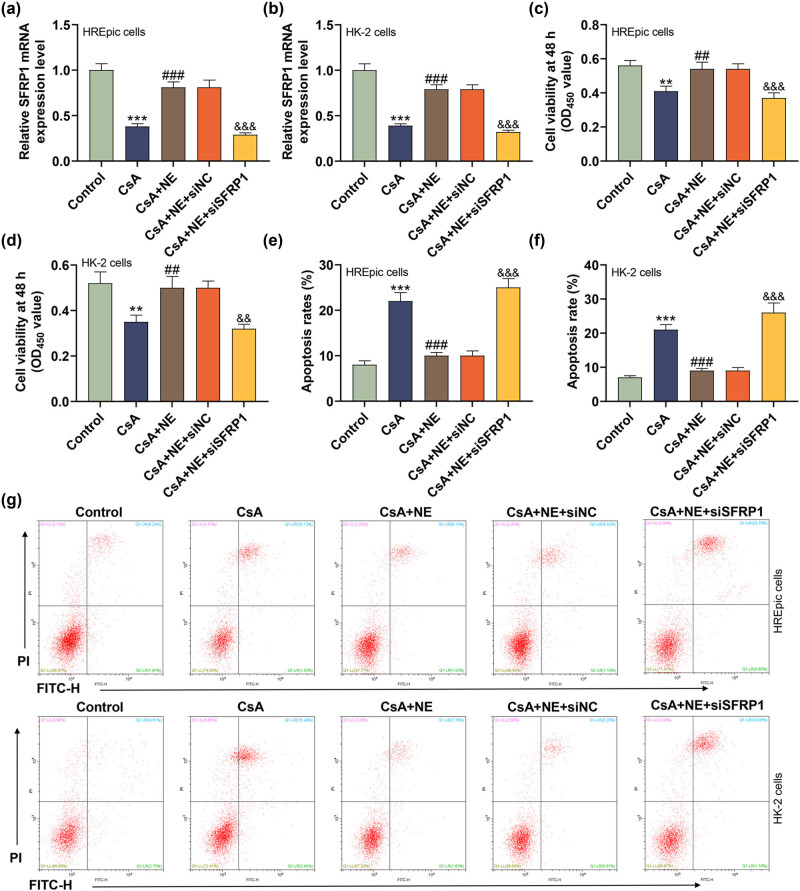
Silencing of SFRP1 reversed the regulation of NE on the SFRP1 level, cell vitality, and apoptosis in HREpic and HK-2 cells induced by CsA. (a) and (b) Effects of SFRP1 knockdown and NE on the SFRP1 level of human renal epithelial cells induced by CsA were examined by qRT-PCR. GAPDH was served as an internal control. (c) and (d) Effects of SFRP1 knockdown and NE on the cell vitality of human renal epithelial cells induced by CsA were examined by CCK-8. (e)–(g) Effects of SFRP1 knockdown and NE on apoptosis of human renal epithelial cells induced by CsA were examined by flow cytometer. All experiments have been performed in triplicate and data were expressed as mean ± SD. ^**^ *P* < 0.01, ^***^ *P* < 0.001 vs control; ^##^ *P* < 0.01, ^###^ *P* < 0.001 vs CsA; ^&&^ *P* < 0.01, ^&&&^ *P* < 0.001 vs CsA + NE + siNC. CsA concentration: 5 μM; NE concentration: 10 μM.

To further explore the effect of SFRP1 on the renal epithelial cells, we transfected siSFRP1 into HREpic and HK-2 cells. As shown in Figure 4a and b, NE intensified the SFRP1 protein level of HREpic and HK-2 cells induced by CsA, while the facilitating effect of NE on the SFRP1 protein level was partially reversed by siSFRP1 (*P* < 0.001). In addition, NE strengthened the vitality of HREpic and HK-2 cells induced by CsA, while siSFRP1 weakened the promoting effect of NE on the vitality of HREpic and HK-2 cells (Figure 4c and d, *P* < 0.01). The cell function experiments illuminated that NE weakened the apoptosis of HREpic and HK-2 cells induced by CsA, which were reversed by siSFRP1 (Figure 4e–g, *P* < 0.01).

## 4 Discussion

The pathogenesis of chronic CsA-induced nephrotoxicity is still unclear. Clinically, it is often characterized by progressive renal function decline, glomerular motility hyaline degeneration, tubular interstitial fibrosis, and interstitial inflammation as pathological features [18]. Its molecular mechanism is extremely complicated and may be related to apoptosis [19], oxidative stress [20], transforming growth factor-β1 [19], inflammatory mediators [21], renin-angiotensin system, and innate immune system [22], which jointly lead to the occurrence of nephrotoxicity. It has been reported that CsA (0.1–40 μM) weakened the cell viability of rat renal mesangial cells and enhanced the levels of pro-apoptotic factors caspase-3, caspase-6, p53, and Bax in a concentration-dependent manner, suggesting that the increased apoptosis of CsA may be one of the mechanisms of promoting CsA-induced nephrotoxicity [23]. *In vivo*, CsA treatment resulted in evident renal histological deterioration and increased the protein expression of renal U-II, which in turn aggravated nephrotoxicity [24]. Ateşşahin et al. demonstrated that CsA led to renal failure in rats, which was characterized by the intensification of plasma creatinine and urea concentrations; CsA also caused renal tubular necrosis, deformation, dilation, and tubulointerstitial fibrosis in rats [25]. Similar to the results of previous studies, we demonstrated that different concentrations’ CsA attenuated the cell vitality but enhanced the LDH activity and apoptosis in HREpic and HK-2 cells. Moreover, our research confirmed for the first time that the SFRP1 level of HREpic and HK-2 cells was notably restrained by different concentrations’ CsA. It was revealed that CsA might trigger nephrotoxicity by repressing the expression of SFRP1.

Bergler et al. established a rat model of kidney transplantation; NE-induced mesenteric resistance artery constriction was largely weakened in rats treated with CsA [5]. Correia et al. demonstrated that medullary interstitial injection of NE could weaken the enhance in diuresis and natriuresis and alleviate renal artery pressure, indicating that NE might restrain these renal hypotensive mechanisms by lessening renal medullary perfusion [26]. Our study complemented the deficiency to explore the role of NE in CsA-induced HREpiC and HK-2 cells. The promotive effects of CsA on the LDH activity and apoptosis of HREpic and HK-2 cells were reversed by NE. Moreover, the inhibitory effect of CsA on the SFRP1 level of HREpic and HK-2 cells was reversed by NE. It was revealed that NE might alleviate CsA-induced nephrotoxicity by enhancing the expression of SFRP1.

In the next research, we transfected siSFRP1 into HREpic and HK-2 cells treated with CsA and NE. As a member of the SFRP family, SFRP1 is a secreted glycoprotein, which can restrain the Wnt/β-catenin signaling pathway by binding to frizzled protein-associated receptors on the surface of the cell membrane, so it is considered to be an inhibitor of the Wnt/β-catenin signaling pathway [27–29]. The studies have confirmed that there was a certain correlation between SFRP1 and the formation of renal interstitial fibrosis, and it was speculated that SFRP1 exerted a critical role in the pathogenesis and development of diabetic nephropathy [12,30]. In this research, we first confirmed the effect of SFRP1 on CsA-triggered nephrotoxicity. NE intensified the SFRP1 level and the cell vitality of HREpic and HK-2 cells induced by CsA, while the elevated effect was partially offset by SFRP1 knockdown. In our current study, we only analyzed that NE alleviates CsA-induced nephrotoxicity by modulating the expression of SFRP1, while the effect of NE on CsA-triggered nephrotoxicity via regulating the upstream and downstream pathways of SFRP1 is the direction of our future research. Moreover, we only analyzed *in vitro* that NE alleviates CsA-triggered nephrotoxicity by regulating SFRP1, which lacks *in vivo* evidence and further clinical verification, which may be the defect of this study.

## 5 Conclusion

To conclude, our research demonstrated that NE might alleviate CsA-induced nephrotoxicity by enhancing the expression of SFRP1. NE may be able to treat CsA-induced nephrotoxicity.
